# Berberine induces FasL-related apoptosis through p38 activation in KB human oral cancer cells

**DOI:** 10.3892/or.2015.3768

**Published:** 2015-01-29

**Authors:** JAE-SUNG KIM, DAHYE OH, MIN-JI YIM, JIN-JU PARK, KYEONG-ROK KANG, IN-A CHO, SUNG-MIN MOON, JI-SU OH, JAE-SEEK YOU, CHUN SUNG KIM, DO KYUNG KIM, SOOK-YOUNG LEE, GYEONG-JE LEE, HEE-JEONG IM, SU-GWAN KIM

**Affiliations:** 1 The Division of Natural Medical Science, College of Health Science, Chosun University, Dong-gu, Gwangju 501-759, Republic of Korea; 2 Oral Biology Research Institute, Chosun University, Dong-gu, Gwangju 501-759, Republic of Korea; 3 Regional Innovation Center for Dental Science and Engineering, Chosun University, Dong-gu, Gwangju 501-759, Republic of Korea; 4 Department of Oral and Maxillofacial Surgery, School of Dentistry, Chosun University, Dong-gu, Gwangju 501-759, Republic of Korea; 5 Department of Prosthodontics, School of Dentistry, Chosun University, Dong-gu, Gwangju 501-759, Republic of Korea; 6 Department of Biochemistry, Rush University Medical Center, Chicago, IL 60612, USA

**Keywords:** berberine, oral cancer, apoptosis, migration, chemotherapy

## Abstract

In the present study, we examined the anticancer properties of berberine in KB oral cancer cells with a specific focus on its cellular mechanism. Berberine did not affect the cell viability of the primary human normal oral keratinocytes that were used as a control. However, the viability of KB cells was found to decrease significantly in the presence of berberine in a dose-dependent manner. Furthermore, in KB cells, berberine induced the fragmentation of genomic DNA, changes in cell morphology, and nuclear condensation. In addition, caspase-3 and -7 activation, and an increase in apoptosis were observed. Berberine was also found to upregulate significantly the expression of the death receptor ligand, FasL. In turn, this upregulation triggered the activation of pro-apoptotic factors such as caspase-8, -9 and -3 and poly(ADP-ribose) polymerase (PARP). Furthermore, pro-apoptotic factors such as Bax, Bad and Apaf-1 were also significantly upregulated by berberine. Anti-apoptotic factors such as Bcl-2 and Bcl-xL were downregulated. Z-VAD-FMK, a cell-permeable pan-caspase inhibitor, suppressed the activation of caspase-3 and PARP. These results clearly indicate that berberine-induced cell death of KB oral cancer cells was mediated by both extrinsic death receptor-dependent and intrinsic mitochondrial-dependent apoptotic signaling pathways. In addition, berberine-induced upregulation of FasL was shown to be mediated by the p38 MAPK signaling pathway. We also found that berberine-induced migration suppression was mediated by downregulation of MMP-2 and MMP-9 through phosphorylation of p38 MAPK. In summary, berberine has the potential to be used as a chemotherapeutic agent, with limited side-effects, for the management of oral cancer.

## Introduction

Oral cancer consists of malignant lesions in the oral cavity and is one of the most common types of cancer worldwide (1,2). Although oral cancer occurs predominantly in individuals aged 50 years and above, there has been a 5–6% increase per year in the number of patients under the age of 45 (3,4). The pathological etiologies of oral cancer are closely associated with mutations in the genes that regulate cell growth, proliferation and apoptosis induced by chronic exposure to carcinogens such as tobacco, alcohol and betel quid (5). Despite improvements and innovations in both diagnostic techniques and therapeutics, surgery often results in loss of function and disfigurement. Furthermore, radiotherapy and the overall survival rate of patients with oral cancer have not improved (6). Therefore, there is an urgent need for the development of clinically relevant, highly effective chemotherapeutic agents with minimal side-effects.

Berberine is a natural isoquinoline alkaloid that is isolated from Oriental herbal plants of the genus *Coptis*. It has been widely investigated due to its biological and pharmacological activities. Recently, the anticancer activity of berberine has been reported in various types of cancer, including brain, lung, esophageal, gastric, colon, skin and liver. This activity is believed to be related to its anti-neoplastic properties leading to the inhibition of cell proliferation, the induction of apoptosis, cell cycle arrest and the inhibition of cell migration (7,8). In a demonstration of the chemotherapeutic promise of berberine, Liu *et al* reported that it exhibited significant cytotoxicity in hepatoma cells, yet showed negligible cytotoxicity to normal cells (9). Furthermore, Hwang *et al* reported that berberine-induced cancer cell apoptosis was mediated by a mitochondrial-dependent intrinsic apoptotic signaling pathway through the activation of caspases and the decreased expression of Bcl-2 and Bcl-xL (10). However, although its potential as a chemotherapeutic agent has been shown, the molecular mechanisms of berberine-induced apoptosis in oral cancer cells are still unknown.

Therefore, the aim of this study was to determine whether berberine has the potential to function as a chemotherapeutic agent by acting on KB oral cancer cells and, at the same time, by not affecting normal cells that originate from the oral cavity. Furthermore, we aimed to evaluate the potential apoptotic effect of berberine and to elucidate the berberine-induced apoptotic signaling pathway in KB cells.

## Materials and methods

### Materials

Anti-FasL, anti-caspase-8, anti-β-actin, anti-Bax, anti-Bad, anti-MMP-2 and anti-MMP-9 were purchased from Santa Cruz Biotechnology, Inc. (Dallas, TX, USA). Anti-cleaved caspase-3, anti-cleaved poly(ADP-ribose) polymerase (PARP), anti-cleaved caspase-9, anti-Bcl-2, anti-Bcl-xL, anti-Bad, anti-Apaf-1, phospho-Erk1/2, total-Erk1/2, phospho-p38, total-p38, phospho-JNK and total JNK were purchased from Cell Signaling (Danvers, MA, USA). ERK chemical inhibitor (PD98059) and p38 chemical inhibitor were purchased from EMD Chemicals (Gibbstown, NJ, USA).

### Cell culture

Normal human oral keratinocytes (NHOKs) were purchased from ScienCell Research Laboratories (Carlsbad, CA, USA). The NHOKs were maintained in Dulbecco’s modified Eagle’s medium (DMEM) containing 10% fetal bovine serum (FBS). The human oral squamous cell carcinoma cell line, KB, was obtained from the American Type Culture Collection (ATCC) and cultured according to the cell culture instructions provided. Briefly, the KB cells were grown in MEM (Gibco, Grand Island, NY, USA) containing 10% FBS (Invitrogen, Carlsbad, CA, USA) at 37°C in an atmosphere containing 5% CO_2_.

### Cell viability assay

Both KB oral cancer cells and NHOKs were seeded at a density of 5×10^5^ cells/well in 96-well plates, and allowed to attach to the well overnight. After incubation, the cultured cells were treated with various concentrations of berberine in triplicate and incubated at 37°C in a 5% humidified CO_2_ incubator for 24 h. MTT was then added to each well and incubation was continued for a further 4 h at 37°C. In order to dissolve the resulting formazan, the cells were resuspended in 200 μl dimethyl sulfoxide (DMSO), and the optical density (OD) of the solution was determined using a spectrometer at an incident wavelength of 570 nm. The experiments were repeated three times, independently. The mean OD ± SD for each group of replicates was calculated. The entire procedure was repeated three times. The inhibitory rate of cell growth was calculated using the equation: % Growth inhibition = [(1 − OD extract treated)/(OD negative control)] × 100.

### Cell survival assay

Cell survival was measured, as previously described (11), using calcein-AM to stain the live cells and ethidium bromide homodimer 1 to stain the dead cells. These reagents were obtained from Molecular Probes (Eugene, OR, USA). For the cell survival assay, KB cells and HNOKs were plated in a chamber slide, incubated with berberine for 24 h, and stained with green calcein-AM and ethidium bromide homodimer 1 according to the manufacturer’s protocol. The cells were then observed and photographed by inverted phase-contrast microscopy (Eclipse TE2000; Nikon Instruments, Melville, NY, USA).

### DNA fragmentation assay

KB oral cancer cells were collected after treatment with berberine (0, 0.1 and 1 μg/ml) and incubation for 24 h, and rinsed three times in phosphate-buffered saline (PBS) at 4°C. The cells were then treated with 100 μl of a cell lysate buffer (1% NP-40, 20 mM EDTA, 50 mM Tris-HCl, pH 7.5) and incubated at 4°C for 10 min, followed by centrifugation at 12,000 × g for 30 min. RNase A was added to the supernatant and incubated at 37°C for 1 h. Proteinase K was then added to the supernatant, which was then incubated at 37°C for 8 h. An equal volume of isopropanol was added and followed by incubation at −80°C for 24 h to precipitate the genomic DNA. The supernatant was removed after centrifugation at 12,000 × g for 15 min at 4°C. The supernatant was allowed to dry naturally and was dissolved in TE buffer, followed by electrophoresis on 1.5% agarose gel. A gel imaging system was used for observation and images were captured.

### Histology

KB cells were collected after treatment with berberine (0, 0.1 and 1 μg/ml) and incubation for 24 h, and rinsed three times in PBS at 4°C. The cells were then fixed with pre-chilled 4% paraformaldehyde for 30 min at 4°C. Hematoxylin and eosin staining was performed in order to observe the morphological changes in the cells. The cells were observed and photographed by inverted phase-contrast microscopy.

### DAPI staining

KB oral cancer cells that had been treated with 0, 0.1 and 1 μg/ml berberine and incubated for 24 h were fixed with 4% paraformaldehyde before washing with PBS. The washed cells were stained with 1 mg/ml [4′,6-diamidino-2-phenylindole dihydrochloride (DAPI); Roche Diagnostics] for 20 min. The nuclear condensation was observed by fluorescence microscopy (Eclipse TE200; Nikon Instruments).

### Caspase-3/-7 activity assay

The apoptotic activity of executioner caspase-3/-7 was determined using the cell-permeable fluorogenic substrate, PhiPhiLux-G_1_D_2_ (OncoImmunin Inc., Gaithersburg, MD, USA), according to the manufacturer’s instructions.

### Flow cytometric analysis

The extent of apoptosis and necrosis was determined using Annexin V-fluorescein isothiocyanate (FITC) and propidium iodide (PI), respectively. The cells were washed twice in PBS and resuspended in a binding buffer (BD Biosciences, San Diego, CA, USA). Annexin V-FITC and PI (BD Biosciences) were added to the cells, which were then incubated in the dark for 15 min. The mixture was then resuspended in 400 ml of binding buffer. The cells were analyzed using a fluorescence-activated cell sorting Calibur flow cytometer (Becton-Dickinson, San Jose, CA, USA). Data analysis was performed using standard Cell Quest software (Becton-Dickinson).

### Immunoblotting

The cell and tissue lysates were prepared using modified radioimmunoprecipitation assay buffer (1 M Tris-HCl, 150 mM NaCl, 1% Triton X-100, 2 mM EDTA) with a protease inhibitor (Sigma, USA) and phosphatase inhibitor cocktail (Sigma-Aldrich, St. Louis, MO, USA). The total protein concentrations of the cell lysates were determined by bicinchoninic acid protein assays (Pierce, Rockford, IL, USA). Equal amounts of the protein were resolved using 10% sodium dodecyl sulfate-polyacrylamide gel electrophoresis and transferred to a nitrocellulose membrane for immunoblotting analyses. After blocking with 5% bovine serum albumin (BSA) in Tris-buffered saline with 0.1% Tween-20 (TBS-T) at room temperature for 1 h, the membranes were incubated sequentially in TBS-T containing 5% BSA and primary antibodies of the corresponding proteins, at 4°C overnight, in accordance with the optimized protocol supplied by the manufacturer. After rinsing with TBS-T, the membranes were incubated with the HRP-conjugated secondary antibody at room temperature for 1 h. The immunoreactivity was visualized using an ECL system (Pierce).

### Gelatin zymography

An equal volume of cell culture supernatant was mixed with non-reducing sample buffer [4% SDS, 0.15 M Tris (pH 6.8) and 20% (vol/vol) glycerol containing 0.05% (wt/vol) bromophenol blue] and resolved on a 10% polyacrylamide gel containing copolymerized 0.2% (1 mg/ml) swine skin gelatin (Sigma). After electrophoresis of the conditioned medium supernatant samples, gels were washed twice, for 15 min each time, with 2.5% Triton X-100. Digestion was carried out by incubating the gel in gelatinase buffer [50 mM Tris-HCl (pH 7.6), 10 mM CaCl_2_, 50 mM NaCl and 0.05% Brij-35] at 37°C for 24 h. The gel was stained with 0.1% Coomassie Brilliant Blue R-250 (GE Healthcare, Piscataway, NJ, USA), and the locations of gelatinolytic activity were revealed as clear bands on a background of uniform light blue staining. After development, gel images were captured and the clear bands were analyzed using ‘ImageJ’ image analysis software (www.imagej.nih.gov).

### Migration assay

To perform the migration assays, KB cells were cultured onto culture inserts (2×0.22 cm^2^; Ibidi, Regensburg, Germany) at 1×10^4^ cells/well. Wounds were introduced by removing the culture inserts after 24 h of incubation. Wound widths were measured using images obtained using an inverted microscope.

### Statistical analysis

Data are reported as the mean ± SD of three individual experiments performed in triplicate. Statistical analysis was carried out using a Student’s t-test, and a p-value <0.05 was considered to indicate a statistically significant result.

## Results

### Berberine induces the cell death of KB oral cancer through apoptosis

**Figure 1:**
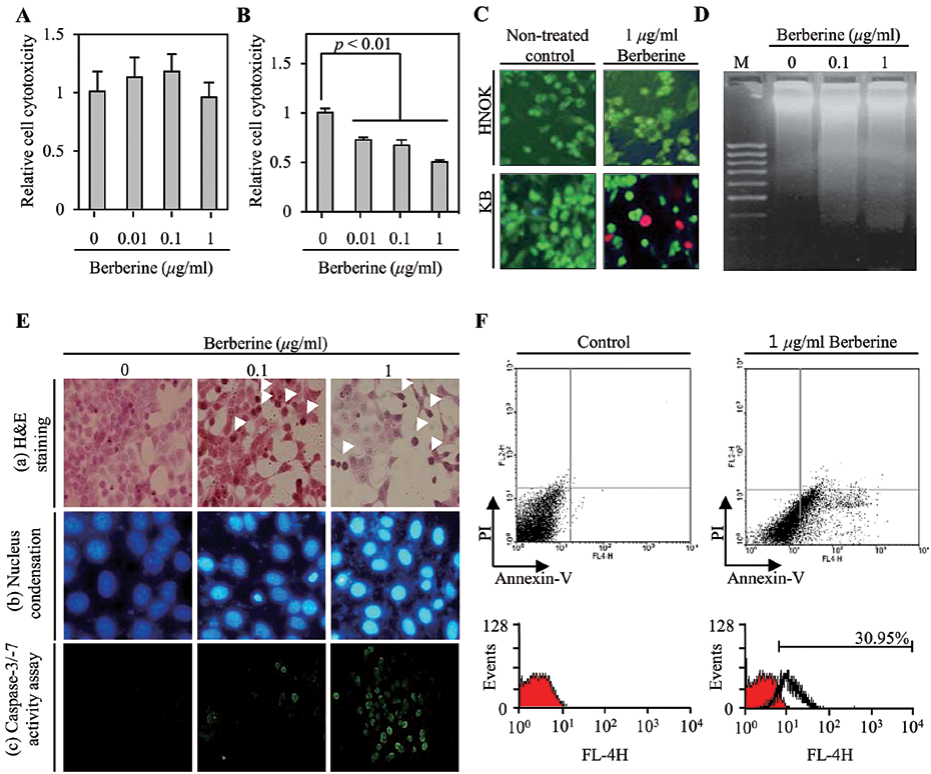
Berberine induces the death of KB oral cancer cells through apoptosis. (A) HNOKs and (B) human KB oral cancer cells were treated with different doses of berberine (0, 0.01, 0.1 and 1 μg/ml) for 24 h. MTT assay was performed to determine cell viability. Data are expressed as the mean ± SD of three independent experiments performed in triplicate (^*^p<0.05 and ^**^p<0.01 compared to the control). (C) Cell survival assay using calcein-AM to stain live cells (green) and ethidium bromide homodimer 1 to stain dead cells (red). (D) Genomic DNA fragmentation products assessed by gel electrophoresis. (E) Formation of apoptotic bodies (a), nuclear condensation (b) and activation of caspase-3 (c). (F) FACS analysis was used to assess the berberine-induced apoptosis of KB oral cancer cells.

To measure the cytotoxicity of berberine in KB oral cancer cells and NHKOs, both cells were treated with various concentrations (0.01, 0.1 and 1 μg/ml) of berberine for 24 h. An MTT assay was then performed to assess the cell viability. As shown in Fig. 1A, berberine did not affect the survival of the primary NHKOs used as normal cells. However, berberine decreased the cell survival rate of the KB oral cancer cells both significantly and in a dose-dependent manner (Fig. 1B). Of particular note, the viability of the KB cells that were treated with 1 μg/ml berberine was decreased by 50% compared to the control. To confirm the berberine-induced cell cytotoxicity in KB cells, microscopy was used to visualize the live and dead cells stained with calcein-AM (green fluorescence) and ethidium homodimer 1 (red fluorescence), respectively. As shown in Fig. 1C, the primary HNOKs incubated with 1 μg/ml berberine for 24 h were stained green due to the cleavage of the membrane permeable calcein-AM by the cytosolic esterase in living cells. Dead cells were stained red by ethidium bromide homodimer 1 and were observed to be a significant presence in the KB oral cancer cells that had been treated with 1 μg/ml berberine. Next, in order to determine whether or not berberine-induced KB oral cancer cell death is related to apoptosis, we measured the extent of representative apoptotic phenomena such as DNA fragmentation, the formation of apoptotic bodies and DNA condensation. As shown in Fig. 1D, DNA fragmentation was observed to a significant degree in the KB oral cancer cells that were treated with 0.1 and 1 μg/ml berberine when compared to the non-treated control. Furthermore, berberine reduced the cell number and altered the typical morphology of KB oral cancer cells in a dose-dependent manner (Fig. 1E-a). Moreover, the number of KB oral cancer cells that had a condensed nucleus was significantly increased by berberine treatment as shown in Fig. 1E-b. To further verify the occurrence of berberine-induced apoptosis in the KB oral cancer cell, we performed a caspase-3/-7 activity assay using PhiPhiLux substrate analyzed using flow cytometry with Annexin V and PI as the stains. As shown in Fig. 1E-c, the activities of caspase-3/-7 increased in a dose-dependent manner in the KB cells that had been treated with berberine. Furthermore, apoptotic population was significantly increased by 30.95% compared to the non-treated control (Fig. 1F). Taken together, these results suggest that berberine induces cell death through apoptosis signaling pathways in KB oral cancer cells.

### Berberine-induced cell death of KB oral cancer is mediated by both extrinsic death receptor-dependent and intrinsic mitochondrial-dependent apoptotic signaling pathways

**Figure 2:**
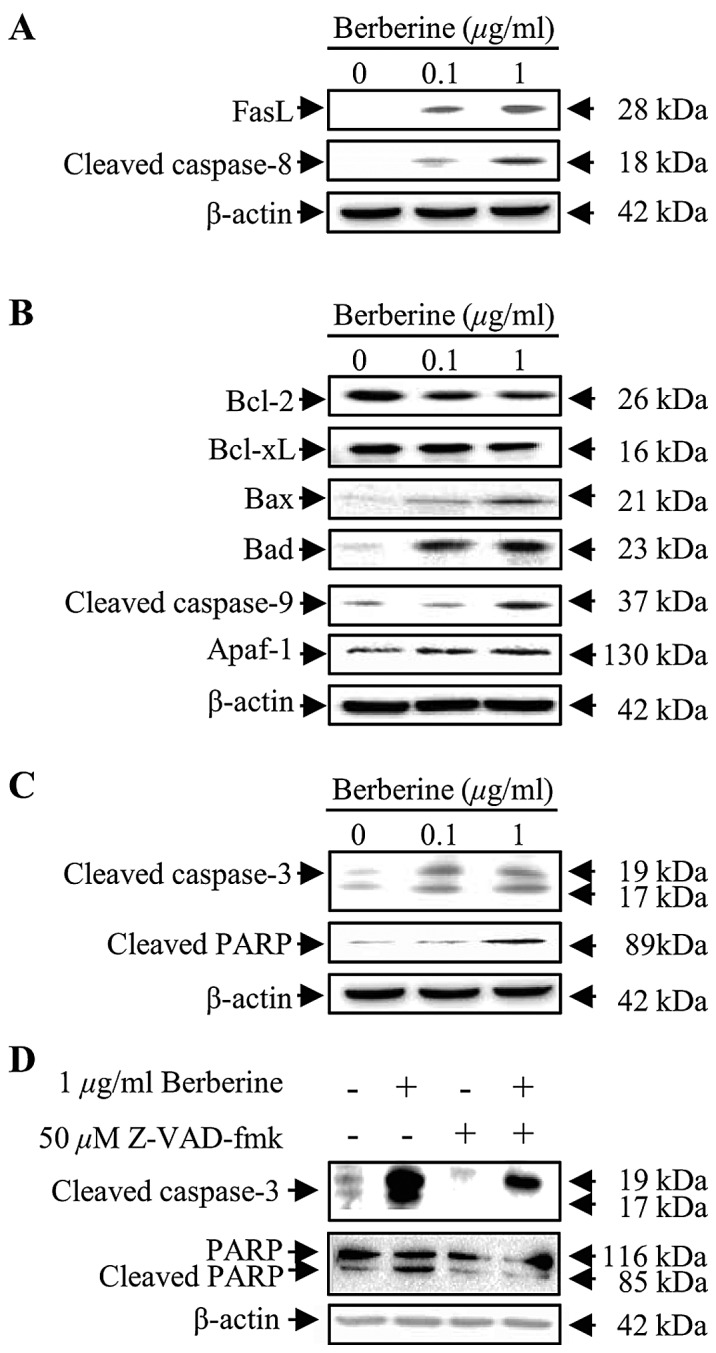
Berberine-induced apoptosis in KB oral cancer cells is mediated by both the extrinsic death receptor-mediated and intrinsic mitochondrial-dependent apoptosis pathways. (A) Berberine-induced extrinsic death receptor-dependent apoptotic signaling pathway; (B) berberine-induced intrinsic mitochondrial-dependent apoptotic signaling pathway. (C) The activation of caspase-3 and PARP via berberine-induced extrinsic death receptor-dependent and intrinsic mitochondrial-dependent apoptosis signaling pathways. (D) Berberine-induced apoptosis of KB oral cancer cells is dependent on caspase activation.

Next, to determine exactly which apoptotic signaling pathways are involved in berberine-induced apoptosis in KB oral cancer cells, we performed western blot analysis using antibodies associated with either the extrinsic death receptor-dependent apoptotic signaling pathway or the intrinsic mitochondrial-dependent apoptotic signaling pathway. As shown in Fig. 2A, FasL (molecular weight 28 kDa), which triggers apoptosis through binding with Fas receptor (FasR) and is known as a member of the death receptor family, was significantly upregulated in a dose-dependent manner in the KB oral cancer cells that were treated with berberine. Furthermore, cleaved caspase-8 (molecular weight 18 kDa), which is a representative death receptor-dependent pro-apoptotic factor, was upregulated by FasL expression. At the same time, berberine decreased the expression of mitochondrial-dependent anti-apoptotic factors such as Bcl-2 (molecular weight 26 kDa) and Bcl-xL (molecular weight 16 kDa) in the KB oral cancer cells. In contrast, mitochondrial-dependent pro-apoptotic factors including Bax (molecular weight 21 kDa), Bad (molecular weight 23 kDa), Apaf-1 (molecular weight 130 kDa) and cleaved caspase-9 (molecular weight 37 kDa) were significantly upregulated following berberine treatment (Fig. 2B). Finally, to induce apoptosis, caspase-3 and PARP were cleaved and activated by upregulation of both extrinsic death receptor-dependent and intrinsic mitochondrial-dependent apoptotic factors in the berberine-treated KB cells (Fig. 2C). Furthermore, to verify whether berberine-induced apoptosis of KB cells is associated with the activation of caspases in both the extrinsic death receptor-dependent and intrinsic mitochondrial-dependent apoptotic signaling pathways, we observed the activation of caspase-3 and PARP in the KB cells that had been treated with 1 μg/ml berberine in either the presence or absence of a cell-permeable pan-caspase inhibitor Z-VAD-FMK (50 μM). As shown in Fig. 2D, in the absence of Z-VAD-FMK, 1 μg/ml berberine induced the activation of caspase-3 and PARP in KB cells. Whereas, the activation of caspase-3 and PARP was significantly suppressed in the KB oral cancer cells treated with 1 μg/ml berberine in addition to Z-VAD-FMK. The observations are consistent with berberine-induced apoptosis of KB cells being mediated by the activation of caspases in both the extrinsic death receptor-dependent and intrinsic mitochondrial-dependent apoptotic signaling pathways.

### Berberine-induced apoptosis of KB oral cancer cells is regulated by expression of death receptor ligand FasL through the p38-MAPK signaling pathway

**Figure 3:**
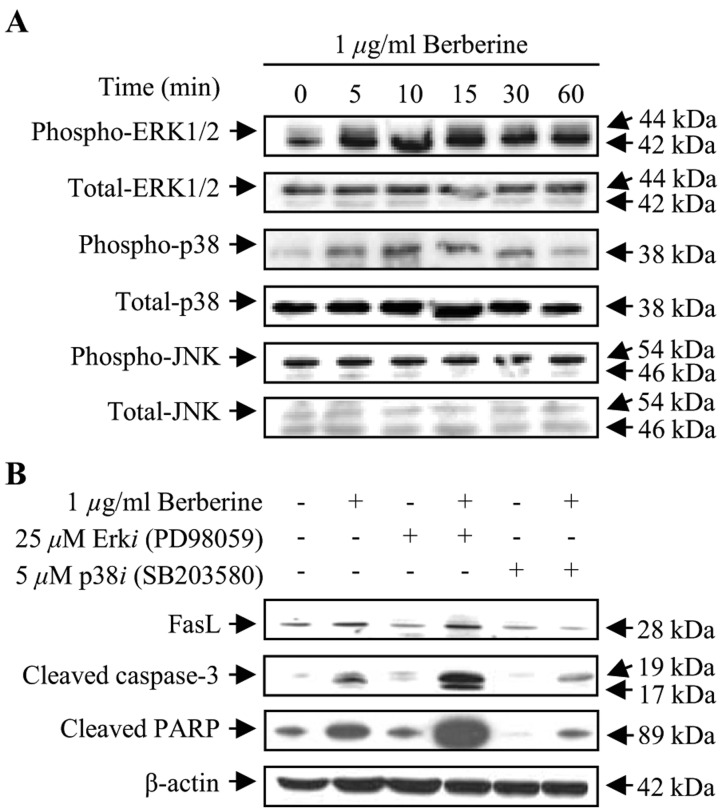
Berberine-induced apoptosis in KB oral cancer cells is triggered by the expression of death receptor ligand FasL via the activation of the p38 MAPK signaling pathway. (A) Berberine induced the phosphorylation of the ERK1/2 and p38 MAPK signaling pathways, yet not the JNK MAPK signaling pathway. (B) Berberine-induced apoptosis was triggered by the expression of FasL via activation of the p38 MAPK signaling pathway.

To understand the signaling pathways involved in the berberine-induced apoptosis of KB oral cancer cells, we examined the activation of mitogen-activated protein kinase (MAPK) subgroups such as ERK1/2, p38 and JNK in response to berberine. As shown in Fig. 3A, the administration of berberine induced the phosphorylation of ERK1/2 (molecular weight 42 and 44 kDa for p42 and p44, respectively) and p38 (molecular weight 38 kDa) MAPK at the earliest time point studied (5 min), and this effect reached a plateau until 60 min post-treatment. However, the phosphorylation of JNK was not observed in the berberine-treated KB oral cancer cells at any of the time points. This suggests that berberine-induced activation of the p38 and ERK1/2 MAPK pathways is the principal pathway involved in the apoptosis mediated by berberine in KB cells. To determine which of these pathways is more principally involved, we inhibited the ERK and p38 MAPK signaling pathways using chemical inhibitors PD98059 and SB203580, respectively. As shown in Fig. 3B, the berberine-induced upregulation of FasL was significantly decreased in the presence of the p38 MAPK-specific chemical inhibitor. In contrast, the ERK1/2 MAPK-specific chemical inhibitor synergistically increased the expression of FasL in the berberine-treated KB cells. Moreover, the observed amounts of both cleaved caspase-3 and cleaved PARP were decreased by the presence of the p38 MAPK-specific chemical inhibitor as well as FasL. Collectively, these findings demonstrate that berberine-induced apoptosis of KB oral cancer cells is regulated by FasL expression via the p38 MAPK signaling pathway.

### Migration of KB oral cancer cells is suppressed by the inhibition of MMP-2/-9 via inhibition of the p38 MAPK signaling pathway

**Figure 4:**
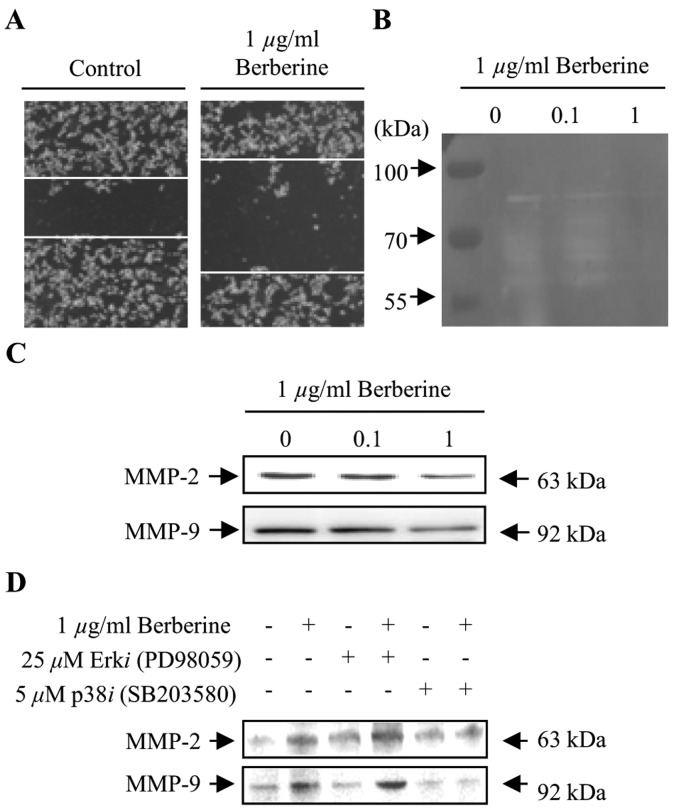
Berberine-induced activation of the p38 MAPK signaling pathway results in suppression of the migration of KB oral cancer cells through the down-regulation of MMP-2 and MMP-3. (A) Berberine suppresses the migration of KB oral cancer cells. Berberine suppresses the (B) activation and (C) expression of matrix metalloproteinases in KB oral cancer cells. (D) Berberine-induced expression of MMP-2 and MMP-9 was regulated by the activation of the p38 MAPK signaling pathway.

Although recent studies have shown that berberine inhibits the migration of various types of cancer cells, the precise effect of berberine on migration and its underlying mechanisms are not fully understood. Therefore, we observed the effect that berberine treatment has on the migration of KB oral cancer cells. As shown in Fig. 4A, the migration of KB cells was significantly decreased in the presence of 1 μg/ml berberine compared to the non-treated control. Therefore, changes in migration-associated factors such as matrix metalloproteinases (MMPs) were examined by carrying out an MMP activation assay using gelatin zymography in conditioned media harvested from KB cells that had been treated with 1 μg/ml berberine for 24 h. As shown in Fig. 4B, the MMP activation was decreased by berberine treatment in a dose-dependent manner. Furthermore, the expression levels of MMP-2 (molecular weight 63 kDa) and MMP-9 (molecular weight 92 kDa), representative MMPs associated with the migration of cancer cells, were downregulated following berberine treatment (Fig. 4C). In addition, the expression of both MMP-2 and MMP-9 was significantly decreased in the KB oral cancer cells that had been co-stimulated with berberine and the p38 MAPK-specific chemical inhibitor compared to those that had been treated with berberine only (Fig. 4D). ERK1/2 MAPK-specific chemical inhibitor synergistically enhanced the expression of both MMP-2 and MMP-9 in the KB cells treated with berberine. Taken together, these results suggest that berberine may suppress the migration of KB oral cancer cells through p38-mediated expressional regulation of MMPs.

## Discussion

Oral cancer is the sixth most common cancer worldwide (1). The 5-year survival rate of patients with oral cancer is less than 50% and this has not improved significantly over the past few decades (12). Oral cancer is defined clinically as cancerous tissue formed in the oral cavity or oropharynx, and it can arise as a primary lesion originating in any of the oral tissues including the soft palate, roof or floor of the mouth, the gums, the tongue, and in other areas of the oral cavity. The clinical symptoms of oral cancer are leukoplakia, erythroplakia, non-healing wounds, sores and tender lesions characterized by painful chewing or swallowing. On the other hand, despite improvements in radiotherapy and chemotherapy for oral cancer, serious side-effects are still encountered. Therefore, an ideal chemotherapeutic agent that effectively suppresses tumorigenesis, leads to minimal side-effects, is inexpensive and easily available is required for clinical oral cancer therapy.

Considerable preclinical and clinical studies have suggested that various plant-derived natural products with a long history of use in Oriental herbal medicine are promising therapeutic agents against a range of cancers. Furthermore, phytochemicals isolated from Oriental herbal plants have shown potential for the prevention of cancer with minimal side-effects, good safety and high efficacy (13). Among the many phytochemicals obtained from Oriental herbal plants, berberine, an isoquinoline derivative alkaloid isolated from the rhizome, roots and stem bark of a number of Oriental herbal plants, such as *Rhizoma coptidis* and *Cortex phellodendri*, has been used as an anti-inflammatory agent (14,15), anti-bacterial compound (16) and antioxidant (17,18). In particular, the anticancer activities of berberine in models of various organs, such as the liver (19), esophagus (20), colon (21), ovaries (22), bladder (23) and breast (24), have been studied. However, the anticancer activity of berberine in oral cancer is not completely understood. Therefore, the present study examined berberine-induced apoptosis and its cellular mechanisms in KB oral cancer cells.

Ideally, chemotherapeutic agents for cancer therapy should not affect adjacent healthy tissues in order to minimize side-effects. In studies of berberine-induced cancer cell-specific cytotoxicity, berberine inhibited the invasion of human cells without eliciting cytotoxicity in healthy cells (9,25). Therefore, we assessed berberine-induced cell cytotoxicity using an MTT assay on primary HNOKs originating from the oral cavity. As shown in Fig. 1A, berberine did not affect the survival of HNOKs at the defined treatment concentration. In contrast, the survival rate of KB oral cancer cells was decreased significantly by berberine in a dose-dependent manner (Fig. 1B). Furthermore, a cell survival assay was performed using image analysis to confirm the results of berberine-induced cell cytotoxicity on both HNOKs and KB cells. According to the data in Fig. 1C, a large number of the KB cells that had been treated with berberine were killed as evidenced by red ethidium bromide homodimer 1 staining. These results suggest that berberine could be used as a potent chemotherapeutic agent with cancer cell-specific effects and minimal side-effects.

Furthermore, these results showed that the anticancer effect of berberine in KB oral cancer cells is closely associated to apoptotic cell death, as shown by the extent of DNA fragmentation, formation of apoptotic bodies and nuclear condensation. Similarly, berberine-induced inhibition of cell proliferation, morphological alteration and nuclear condensation were also observed in the human lung cancer cell lines, A549 and H1299 (25). Therefore, a caspase-3/-7 activity assay was performed using the cell-permeable fluorogenic substrate PhiPhiLux staining. As shown in Fig. 1E-c, activated caspase-3/-7 was observed in the berberine-treated KB cells. Caspase-3, a representative pro-apoptotic factor, was activated in the end stages of both the extrinsic death receptor-mediated and the mitochondrial-dependent apoptotic signaling pathways (Fig 2C). Furthermore, the apoptotic population stained using Annexin-5 (that binds to the surface of apoptotic cells) increased in a time-dependent manner in the KB oral cancer cells that had been treated with berberine (Fig. 1F). Therefore, berberine-induced cancer-specific cell death is mediated via the apoptotic signaling pathway.

According to a recent report, berberine-induced cell death is mediated via the intrinsic mitochondrial-dependent apoptotic signaling pathway in a range of cancers, including liver (26,27) and breast cancer (28). To verify the apoptotic signaling pathway in berberine-treated KB, the activation and/or expression of apoptotic factors associated with apoptosis was assessed by immunoblotting. As shown in Fig. 2, death receptor ligand FasL, which initiates the extrinsic death receptor-mediated apoptotic signaling pathway, was upregulated significantly by berberine treatment in the KB oral cancer cells. Pro-caspase-8 was then activated via interaction with Fas-interacting protein FADD. In addition, activated caspase-8 induced the cleavage of pro-caspase-3 to activate poly(ADP ribose) polymerase (PARP). Finally, activated PARP induced DNA fragmentation via single-strand DNA breaking in the nucleus of cancer cells. Furthermore, Bcl-2 and Bcl-xL, anti-apoptotic factors associated with the intrinsic mitochondrial-dependent apoptotic signaling pathway, were downregulated significantly by berberine treatment in the KB oral cancer cells. Both the downregulation of the anti-apoptotic factors and upregulation of both pro-apoptotic factors and tumor suppression factors triggered the release of cytochrome *c* from the mitochondria. In addition, apoptotic protease activating factor-1 (Apaf-1) was upregulated significantly by berberine in the KB oral cancer cells. Both the released cytochrome *c* and upregulated Apaf-1 induced the activation of caspase-9 leading to the activation of caspase-3 and PARP. Recent studies of berberine-induced apoptosis in oral cancer reported that berberine induced apoptosis by promoting the expression of caspase-8, -9 and -3 in the human tongue squamous carcinoma cell line, SCC-4 (29). Ho *et al* suggested that berberine-induced apoptosis was mediated by both apoptotic signaling pathways (29). Similarly, Lin *et al* reported that berberine induced apoptosis in human HSC-3 oral cancer cells via activation of both the death receptor-mediated and the mitochondrial pathways (30). Therefore, these findings suggest that berberine-induced KB cell death is mediated by both the extrinsic death receptor-mediated pathway and the intrinsic mitochondrial-dependent apoptotic signaling pathway. Upregulated FasL, however, not only triggers the extrinsic death receptor-mediated apoptotic signaling pathway, but also initiates the intrinsic mitochondria-dependent apoptotic pathway via the cleavage of Bid to tBid by activated caspase-8. Although the cleavage of Bid to tBid was not observed in the present study, Bax, a downstream, pro-apoptotic factor of tBid, was upregulated significantly by berberine treatment (Fig. 2B). This suggests that activated caspase-8 in the extrinsic apoptosis signaling pathway can affect the intrinsic mitochondrial-dependent apoptotic signaling pathway in KB oral cancer cells.

Although there have been a large number of *in vitro* studies on the cellular function of MAPKs, the biological connection between berberine-induced apoptosis and the MAPK signaling pathway is largely unknown. Recently, Zheng *et al* (31), reported that berberine-induced apoptosis of human lung adenocarcinoma was mediated by activation of the p38 MAPK signaling pathway. Similarly, our results showed that berberine induced the phosphorylation of both ERK1/2 and p38 MAPK in the KB oral cancer cells (Fig. 3). Furthermore, berberine-induced apoptosis was mediated by the expression of death receptor ligand FasL, which induces apoptosis in various cancer cells, through the activation of the p38 MAPK signaling pathway, yet not the ERK1/2 MAPK signaling pathway. Even though we did not observe a connection at the cellular level between the activation of the p38 MAPK signaling pathway and the expression of p53 in the present study, Zheng *et al* (31), reported that berberine upregulates the tumor suppressor p53 through the activation of the p38 MAPK signaling pathway and reduces the cancer cell proliferation and induces cell cycle arrest. Therefore, our data suggest that berberine may induce cell cycle arrest and the inhibition of cell proliferation in KB oral cancer cells.

One of main characteristics of malignant cancer cells is invasion and metastasis. Recently, many studies found that berberine suppressed the migration of various types of cancer cells such as T24 bladder (32) and human colon cancer (33), melanoma (34) and human SCC-4 tongue squamous cancer cells (35), yet the cellular physiological mechanisms of berberine involved in the migration of KB oral cancer cells have yet to be reported. However, the migration and invasion of cancer cells are closely associated with expression of MMPs, which are required for remodeling of the extracellular matrix. MMP-9, a downstream target molecule of the MAPK subfamily, is particularly associated with cancer cell invasion. Furthermore, the expression of MMP-2 is associated with tumor invasion and metastasis. Therefore, the expressional suppression of MMP-2 and MMP-9 may result in the inhibition of migration in cancer cells. In the present study, we demonstrated that berberine suppressed the migration of KB oral cancer cells through the downregulation of both MMP-2 and MMP-9 (Fig. 4). Recently, Ho *et al* (35*)* reported that berberine suppressed *in vitro* migration and invasion of human SCC-4 tongue squamous cancer cells through the inhibition of MMP-2 and MMP-9 and was regulated by nuclear factor-κB (NFκB). In contrast, in the KB oral cancer cells that were treated with berberine, the expression of MMP-2 and MMP-3 was regulated by the p38 MAPK signaling pathway (Fig. 4D).

In summary, our data demonstrated that berberine-induced apoptosis of KB oral cancer cells was triggered by the expression of FasL via the activation of the p38 MAPK signaling pathway and was mediated by both extrinsic death receptor-dependent and intrinsic mitochondrial-dependent apoptotic signaling pathways. Furthermore, berberine-induced suppression of migration in KB cells was mediated by the downregulation of MMP-2 and MMP-7 through the activation of the p38 MAPK signaling pathway. Indeed, berberine may have great potential for the future treatment of oral cancer, and its potent anticancer properties highlight its promise as a chemotherapeutic agent.

## References

[1] Sunil PM, Ramachandran CR, Arulmoli, Devi S, Soma SV (2011). Cytogenetic alterations in oral squamous cell carcinoma detected by karyotyping (g-banding). OMPJ.

[2] Siegel R, Naishadham D, Jemal A (2012). Cancer statistics, 2012. CA Cancer J Clin.

[3] Scully C (2011). Oral cancer aetiopathogenesis; past, present and future aspects. Med Oral Patol Oral Cir Bucal.

[4] Llewellyn CD, Johnson NW, Warnakulasuriya KA (2001). Risk factors for squamous cell carcinoma of the oral cavity in young people - a comprehensive literature review. Oral Oncol.

[5] Sugerman PB, Savage NW (1999). Current concepts in oral cancer. Aust Dent J.

[6] Gupta S, Kong W, Peng Y, Miao Q, Mackillop WJ (2009). Temporal trends in the incidence and survival of cancers of the upper aerodigestive tract in Ontario and the United States. Int J Cancer.

[7] Tang J, Feng Y, Tsao S, Wang N, Curtain R, Wang Y (2009). Berberine and Coptidis rhizoma as novel antineoplastic agents: a review of traditional use and biomedical investigations. J Ethnopharmacol.

[8] Chen TC, Lai KC, Yang JS (2009). Involvement of reactive oxygen species and caspase-dependent pathway in berberine-induced cell cycle arrest and apoptosis in C6 rat glioma cells. Int J Oncol.

[9] Liu B, Wang G, Yang J, Pan X, Yang Z, Zang L (2011). Berberine inhibits human hepatoma cell invasion without cytotoxicity in healthy hepatocytes. PLoS One.

[10] Hwang JM, Kuo HC, Tseng TH, Liu JY, Chu CY (2006). Berberine induces apoptosis through a mitochondria/caspases pathway in human hepatoma cells. Arch Toxicol.

[11] Kim JS, Ellman MB, An HS, van Wijnen AJ, Borgia JA, Im HJ (2010). Insulin-like growth factor 1 synergizes with bone morphogenetic protein 7-mediated anabolism in bovine intervertebral disc cells. Arthritis Rheum.

[12] Brinkman BM, Wong DT (2006). Disease mechanism and biomarkers of oral squamous cell carcinoma. Curr Opin Oncol.

[13] Sundin T, Peffley DM, Hentosh P (2013). Disruption of an hTERT-mTOR-RAPTOR protein complex by a phytochemical perillyl alcohol and rapamycin. Mol Cell Biochem.

[14] Jiang Q, Liu P, Wu X (2011). Berberine attenuates lipopolysaccha-ride-induced extracelluar matrix accumulation and inflammation in rat mesangial cells: involvement of NF-κB signaling pathway. Mol Cell Endocrinol.

[15] Lin K, Liu S, Shen Y, Li Q (2013). Berberine attenuates cigarette smoke-induced acute lung inflammation. Inflammation.

[16] Bandyopadhyay S, Patra PH, Mahanti A (2013). Potential antibacterial activity of berberine against multi drug resistant enterovirulent Escherichia coli isolated from yaks (Poephagus grunniens) with haemorrhagic diarrhoea. Asian Pac J Trop Med.

[17] Tan Y, Tang Q, Hu BR, Xiang JZ (2007). Antioxidant properties of berberine on cultured rabbit corpus cavernosum smooth muscle cells injured by hydrogen peroxide. Acta Pharmacol Sin.

[18] Zhou JY, Zhou SW (2011). Protective effect of berberine on anti-oxidant enzymes and positive transcription elongation factor b expression in diabetic rat liver. Fitoterapia.

[19] Tan YL, Goh D, Ong ES (2006). Investigation of differentially expressed proteins due to the inhibitory effects of berberine in human liver cancer cell line HepG2. Mol Biosyst.

[20] Iizuka N, Miyamoto K, Okita K (2000). Inhibitory effect of Coptidis Rhizoma and berberine on the proliferation of human esophageal cancer cell lines. Cancer Lett.

[21] Fukuda K, Hibiya Y, Mutoh M, Koshiji M, Akao S, Fujiwara H (1999). Inhibition by berberine of cyclooxygenase-2 transcriptional activity in human colon cancer cells. J Ethnopharmacol.

[22] Marverti G, Ligabue A, Lombardi P (2013). Modulation of the expression of folate cycle enzymes and polyamine metabolism by berberine in cisplatin-sensitive and -resistant human ovarian cancer cells. Int J Oncol.

[23] Yan K, Zhang C, Feng J (2011). Induction of G1 cell cycle arrest and apoptosis by berberine in bladder cancer cells. Eur J Pharmacol.

[24] Ma X, Zhou J, Zhang CX (2013). Modulation of drug-resistant membrane and apoptosis proteins of breast cancer stem cells by targeting berberine liposomes. Biomaterials.

[25] Fu L, Chen W, Guo W (2013). Berberine targets AP-2/hTERT, NF-κB/COX-2, HIF-1α/VEGF and cytochrome-c/caspase signaling to suppress human cancer cell growth. PLoS One.

[26] Yip NK, Ho WS (2013). Berberine induces apoptosis via the mitochondrial pathway in liver cancer cells. Oncol Rep.

[27] Wang N, Feng Y, Zhu M (2010). Berberine induces autophagic cell death and mitochondrial apoptosis in liver cancer cells: the cellular mechanism. J Cell Biochem.

[28] Patil JB, Kim J, Jayaprakasha GK (2010). Berberine induces apoptosis in breast cancer cells (MCF-7) through mitochondrial-dependent pathway. Eur J Pharmacol.

[29] Ho YT, Lu CC, Yang JS (2009). Berberine induced apoptosis via promoting the expression of caspase-8, -9 and -3, apoptosis-inducing factor and endonuclease G in SCC-4 human tongue squamous carcinoma cancer cells. Anticancer Res.

[30] Lin CC, Yang JS, Chen JT (2007). Berberine induces apoptosis in human HSC-3 oral cancer cells via simultaneous activation of the death receptor-mediated and mitochondrial pathway. Anticancer Res.

[31] Zheng F, Tang Q, Wu J (2014). p38α MAPK-mediated induction and interaction of FOXO3a and p53 contribute to the inhibited-growth and induced-apoptosis of human lung adenocarcinoma cells by berberine. J Exp Clin Cancer Res.

[32] Yan L, Yan K, Kun W (2013). Berberine inhibits the migration and invasion of T24 bladder cancer cells via reducing the expression of heparanase. Tumour Biol.

[33] Park JJ, Seo SM, Kim EJ (2012). Berberine inhibits human colon cancer cell migration via AMP-activated protein kinase-mediated downregulation of integrin β1 signaling. Biochem Biophys Res Commun.

[34] Singh T, Vaid M, Katiyar N, Sharma S, Katiyar SK (2011). Berberine, an isoquinoline alkaloid, inhibits melanoma cancer cell migration by reducing the expressions of cyclooxygenase-2, prostaglandin E_2_ and prostaglandin E_2_ receptors. Carcinogenesis.

[35] Ho YT, Yang JS, Li TC (2009). Berberine suppresses in vitro migration and invasion of human SCC-4 tongue squamous cancer cells through the inhibitions of FAK, IKK, NF-κB, u-PA and MMP-2 and -9. Cancer Lett.

